# The Alzheimer’s Knowledge Base: A Knowledge Graph for Alzheimer Disease Research

**DOI:** 10.2196/46777

**Published:** 2024-04-18

**Authors:** Joseph D Romano, Van Truong, Rachit Kumar, Mythreye Venkatesan, Britney E Graham, Yun Hao, Nick Matsumoto, Xi Li, Zhiping Wang, Marylyn D Ritchie, Li Shen, Jason H Moore

**Affiliations:** 1 Institute for Biomedical Informatics Perelman School of Medicine University of Pennsylvania Philadelphia, PA United States; 2 Center of Excellence in Environmental Toxicology Perelman School of Medicine University of Pennsylvania Philadelphia, PA United States; 3 Department of Biostatistics, Epidemiology and Informatics Perelman School of Medicine University of Pennsylvania Philadelphia, PA United States; 4 Graduate Group in Genomics and Computational Biology Perelman School of Medicine University of Pennsylvania Philadelphia, PA United States; 5 Department of Genetics Perelman School of Medicine University of Pennsylvania Philadelphia, PA United States; 6 Medical Scientist Training Program Perelman School of Medicine University of Pennsylvania Philadelphia, PA United States; 7 Department of Computational Biomedicine Cedars-Sinai Medical Center Los Angeles, CA United States

**Keywords:** Alzheimer disease, knowledge graph, knowledge base, artificial intelligence, drug repurposing, drug discovery, open source, Alzheimer, etiology, heterogeneous graph, therapeutic targets, machine learning, therapeutic discovery

## Abstract

**Background:**

As global populations age and become susceptible to neurodegenerative illnesses, new therapies for Alzheimer disease (AD) are urgently needed. Existing data resources for drug discovery and repurposing fail to capture relationships central to the disease’s etiology and response to drugs.

**Objective:**

We designed the Alzheimer’s Knowledge Base (AlzKB) to alleviate this need by providing a comprehensive knowledge representation of AD etiology and candidate therapeutics.

**Methods:**

We designed the AlzKB as a large, heterogeneous graph knowledge base assembled using 22 diverse external data sources describing biological and pharmaceutical entities at different levels of organization (eg, chemicals, genes, anatomy, and diseases). AlzKB uses a Web Ontology Language 2 ontology to enforce semantic consistency and allow for ontological inference. We provide a public version of AlzKB and allow users to run and modify local versions of the knowledge base.

**Results:**

AlzKB is freely available on the web and currently contains 118,902 entities with 1,309,527 relationships between those entities. To demonstrate its value, we used graph data science and machine learning to (1) propose new therapeutic targets based on similarities of AD to Parkinson disease and (2) repurpose existing drugs that may treat AD. For each use case, AlzKB recovers known therapeutic associations while proposing biologically plausible new ones.

**Conclusions:**

AlzKB is a new, publicly available knowledge resource that enables researchers to discover complex translational associations for AD drug discovery. Through 2 use cases, we show that it is a valuable tool for proposing novel therapeutic hypotheses based on public biomedical knowledge.

## Introduction

### Background

Alzheimer disease (AD) is a progressive, neurodegenerative disease affecting an estimated 6.5 million Americans aged ≥65 years and represents a significant clinical, economic, and emotional burden worldwide [1]. AD is often cited as one of the greatest health care problems of the 21st century, particularly in high-income nations with an increasing proportion of older adults. Despite its societal impact, effective pharmaceutical treatments for AD remain notoriously elusive. The US Food and Drug Administration has approved 5 drugs for the treatment of AD, 4 of which (donepezil, rivastigmine, galantamine, and memantine) only temporarily treat symptoms but do not alter the overall progression of the disease [2], whereas the fifth (aducanumab) is highly controversial in terms of evidence of effectiveness and its safety profile [3]. AD researchers have prioritized the discovery and approval of new therapies for the disease both in terms of newly discovered compounds and by repurposing drugs that are already approved to treat other (non-AD) human diseases.

AD is associated with substantial changes in pathology, including the presence of neuritic plaques associated with the amyloid-β protein, extracellular deposition of amyloid-β, and neurofibrillary tangles. Previous research has shown that these neuropathological changes begin to occur years before clinical symptoms are apparent [4,5]. Despite decades of research, why this pathology begins to develop remains largely unknown [6]. Current consensus is that AD risk is multifactorial. The most well-established risk factors include age; family history; and certain genetic factors, especially the presence of the σ4 allele of the *apolipoprotein E* gene, which is involved in fat metabolism and cholesterol transport. However, the exact mechanism through which these factors—including *APOE*-σ4 presence—cause or contribute to AD risk is unknown [7].

Of the many techniques used in AD therapeutics research, there is a wealth of computer-aided approaches that leverage recent advances in bioinformatics, epidemiology, artificial intelligence (AI), and machine learning (ML). For example, Rodriguez et al [8] developed an ML framework to assess gene lists constructed by differential gene expression data in response to drug treatment to determine whether those drugs would be candidates for repurposing in AD. Tsuji et al [9] used an autoencoder neural network to perform dimensionality reduction of a high-density protein interaction network to identify new possible drug targets and then found drugs associated with those targets. Genome-wide association studies have long been used for the identification of genes that confer AD risk, particularly for rare genes or genes with small (but statistically significant) contributions to disease risk [10].

**Figure 1:**
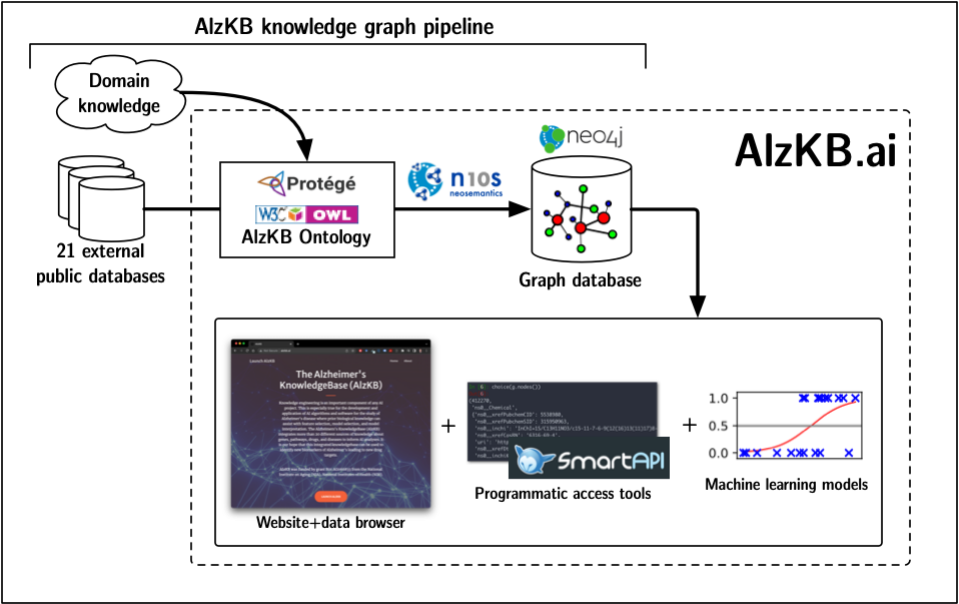
Schematic overview of the Alzheimer’s Knowledge Base (AlzKB).

In this paper, we describe the design and deployment of a major new knowledge resource for computational AD research—named The Alzheimer’s Knowledge Base (AlzKB) [11]—with a particular focus on drug discovery and drug repurposing. The overall structure and contents of AlzKB are summarized in Figure 1. At its core, AlzKB consists of a large, heterogeneous graph database describing entities related to AD at multiple levels of biological organization, with rich semantic relationships describing how those entities are linked to one another. To demonstrate its value, we present two data-driven analyses involving ML on AlzKB’s knowledge graph: (1) predicting Parkinson disease (PD) genes that may also be associated with AD and (2) generating and explaining drug repurposing hypotheses for treating AD, both of which replicate existing knowledge while proposing entirely novel directions for future experimental validation. AlzKB is free, open source, and publicly available [11] and consists entirely of publicly sourced knowledge integrated from 22 diverse web-based biomedical databases. We hypothesized that the relationships and entities in AlzKB contain valuable knowledge that cannot be effectively captured in existing data resources, with the additional advantage of improving the explainability of new predictions.

### Existing Graph-Based Approaches to AD Research

Due to the increased popularity and success of analyses using integrated knowledge, previous efforts have used knowledge graphs in AD research for a variety of purposes, including drug repurposing [12-14] and gene identification [15] and as general informational resources [16]. Similar to AlzKB, these bodies of work draw from a variety of sources to construct the underlying knowledge graphs, including scientific literature and formally structured biomedical databases. Some, including the Alzheimer Disease Knowledge Graph [14] and the Heterogeneous network-based data set for AD [16], have been released as publicly accessible resources similar to AlzKB. Other studies have used existing resources not specifically intended for AD research (such as the Semantic MEDLINE Database [13]) to answer questions related to AD. To our knowledge, AlzKB is the largest graph-based knowledge representation that focuses solely on AD and draws from the greatest number of source databases. For comparison, the next largest AD-specific knowledge graph that we are aware of is AD-KG, which contains 30,729 nodes and 398,544 edges (compared to AlzKB’s 118,902 nodes and 1,309,527 edges). Our emphasis on merging similar nodes or edges and cleaning the graph structure using an underlying biomedical ontology reduces the amount of noise that tends to be associated with many different node or edge types in a single graph, enabling more robust inference about relationships in AD, especially when used with emerging graph ML algorithms. Furthermore, AlzKB offers a public, web interface that allows for easy access and application to new research questions, whereas existing resources have either restricted access or are entirely unavailable for reuse. Given the challenge of identifying new or repurposed drugs for etiologically complex diseases such as AD, AlzKB represents a major step forward by improving both quantitatively and structurally on existing resources.

## Methods

### AlzKB Ontology

Graph databases are renowned for their flexibility in representing data that do not conform to a rigid, tabular structure, but this comes at the expense of implicitly enforcing consistency and semantic standardization [17]. To mitigate this issue, we designed a Web Ontology Language (OWL) 2 ontology—describing the types of entities relevant to AD and treatment of AD, as well as the types of relationships that link those entities—that serves as a *template* for nodes and edges in the knowledge graph. Ontologies (including OWL 2 ontologies) are formal representations of knowledge that are frequently used in biomedicine to computationally structure, retrieve, and make inferences about knowledge within a domain of interest [18]. Briefly, as many of the components of a graph database have a 1-to-1 correspondence with components of an OWL 2 ontology (eg, OWL 2 classes are equivalent to graph database node labels, and OWL 2 object properties are equivalent to edge types in a graph database), it is possible to populate the ontology using biomedical knowledge and translate the contents of the populated ontology into an equivalent graph database. Therefore, enforcing consistency in the ontology becomes equivalent to enforcing consistency in the graph database.

We constructed the ontology manually using the Protégé ontology editor (version 5.5.0; Stanford Center for Biomedical Informatics Research) [19] following an iterative process guided by expert domain knowledge. First, we prototyped a class hierarchy containing the types of nodes (eg, gene, disease, pathway, and drug) desired in the knowledge base. We then annotated these classes with data properties (eg, drugs can be assigned a property value corresponding to molecular weight) and object properties (relationship types that link 2 entities, such as “drug treats disease”). A thorough description of the components of OWL 2 ontologies is provided by Hitzler et al [20]. Finally, we placed restrictions on the ontology to reflect biology and clinical practice. For example, we specified restrictions stating that all pathways must contain one or more genes or that all drugs in the knowledge base must have a valid DrugBank ID. We repeated these steps several times, making revisions on previous iterations until several domain experts agreed that the semantic contents of the ontology were consistent with current AD knowledge and systems biology processes involved in AD etiology. After collecting the data sources used to populate the ontology (see the following section), we included additional data properties corresponding to identifiers in those source databases, enabling data provenance and facilitating both interoperability and validation. The final ontology structure consists of entity types involved in AD etiology (modeled as OWL 2 classes), types of semantic relationships that can link those entity types (modeled as OWL 2 object properties), and properties that can be annotated onto entities of specific types (modeled as OWL 2 data properties). Both before and after populating the ontology with individuals (see the *Implementing AlzKB* section), we validated its contents and structure by running FaCT++—an ontology inference engine that identifies errors by evaluating all assertions in the ontology against the ontology’s class or property hierarchy and other restrictions [21].

### Collecting and Assembling Third-Party Data Sources

Using the AlzKB ontology’s class hierarchy as a starting point, we determined a set of the most important entity types to include in the first release of the knowledge base. For example, we prioritized inclusion of entities representing diseases (specifically AD and its various subtypes), genes, and drugs, among others. Similarly, we identified important relationship types (eg, “DRUG_BINDS_GENE” or “GENE_ASSOCIATED_WITH_DISEASE”) to include in the knowledge base. For each of these entity and relationship types, we identified a third-party, public data source that would serve as a collection of “ground truth knowledge” for that entity or relationship type. In the assembled knowledge base, there is roughly a 1-to-1 correspondence between a data record in the original “ground truth” data source and its corresponding entity or relationship in AlzKB, with some important exceptions. For example, we made the decision to only include neurological diseases in AlzKB rather than all diseases described in the “ground truth” data source (in this case, the Disease Ontology). We also identified instances in which properties from additional data sources could be used to augment the “ground truth” entities. For example, while DrugBank is used to specify the drugs described in AlzKB, we also used fields from Distributed Structure-Searchable Toxicity and PubChem to augment the properties annotated onto drugs (such as molecular weight, chemical fingerprint, and synonyms).

### Implementing AlzKB

We populated the ontology by sequentially carrying out the following steps:

1. Import distinct entities from each data source corresponding to the corresponding ontology class and define those entities as ontology individuals (ie, instances of that class). For example, the drug memantine is defined as an instance of the ontology class Drug.
2. Populate data properties for all instances of each ontology class using data from relevant sources. For example, memantine is annotated with the Chemical Abstracts Service Registry number 19982-08-2.
3. Populate object properties as the semantic relationships linking pairs of entities using the appropriate data source. For example, an object property of type “DRUG_TREATS_DISEASE” links memantine to the instance of Disease named Alzheimer’s Disease.

After populating the AlzKB ontology with entities, relationships, and data properties, we serialized the ontology into the Resource Description Framework (RDF) or XML graph data format, which is compatible with modern graph database software as an input format. A complete list of the data sources used in AlzKB at the time of writing is provided in Table 1. We then populated a Neo4j graph database (version 4.4.5; Neo4j, Inc) [22] with the contents of the RDF or XML file using the neosemantics library [23], which parses the RDF data, inserting semantic triples into the graph database corresponding to each entity or relationship. Finally, we stripped the newly populated graph database of unnecessary artifacts that are components of the OWL 2 standard, leaving only nodes, relationships, and properties defined within the hierarchy. For the publicly hosted version of AlzKB, we created a web server that hosts both the static AlzKB website (containing information, documentation, and use details) and the Neo4j graph database, which is available by navigating to a subdomain [24] of the main website [11]. For reproducibility, this entire pipeline (including mappings to source databases) is provided as a single Python script available on GitHub (the most recent version) [25] or Zenodo (an archived version of the code at the time of publication) [26].

**Table 1:** Third-party public data sources used in the Alzheimer’s Knowledge Base (AlzKB), which data elements were used from them, and total size of the data source (counts of entities of relevant data types only)^a^.

Data source	Use in AlzKB	Size (number of entities)
AOP-DB^b^ [27]	Adverse outcome pathways and chemical-gene associations	1,207,456
Bgee^c^ [28]	Tissue-specific gene expression data; only human gene expression data were used in AlzKB	9,093,494
Disease Ontology^c^ [29]	Human diseases—only AD^d^, subtypes of AD, and related neurodegenerative diseases were included in AlzKB	8043
DisGeNET [30]	Diseases, genes, and disease-gene associations with scores representing levels of evidence; only AD and related neurodegenerative terms were used for diseases	51,841
DrugBank [31]	On-market and experimental pharmaceutical drugs	15,550
EPA^e^ DSSTox^f^ [32]	Chemical toxicity data—filtered in AlzKB to drugs contained in DrugBank	1,200,059
EPA ACToR^g^ [33]	Chemical toxicity data—filtered in AlzKB to drugs contained in DrugBank	504,871
Gene Ontology^c^ [34,35]	Biological processes, molecular functions, and cellular components	42,950
GWAS^h^ Catalog^c^ [36]	Gene-disease associations	60,071
Hetionet [37]	Graph modeling and entity resolution (for data sources marked with footnote indicator “c”)	47,031
Human Reference Protein Interactome Mapping Project^c^ [38]	Human protein-protein interactions (modeled as gene-gene interactions)	9094
LINCS^i^ L1000^c^ [39]	Human differential gene expression data	7467 genes
NCBI^j^ MeSH^k^ [40]	Clinical and biomedical concepts (annotated to various node types)	Approximately 27,000
NCBI Entrez Gene [41]	Human genes and gene synonyms	62,407
Pathway Interaction Database^c^ [42]	Pathways and gene-pathway membership	223
PharmacotherapyDB^c^ [43]	Drug indications for human diseases	698
PubChem [44]	Chemical structures and identifiers—only chemicals in DrugBank were included in AlzKB	115,067,800
Reactome pathway database^c^ [45]	Pathways and gene-pathway membership	1341
SIDER^c,l^ [46]	Drug side effects (modeled as diseases)	5868
TISSUES^c^ [47]	Tissue-specific gene expression data	—^m^
Uberon^c^ [48]	Human anatomical structures	402
WikiPathways^c^ [49]	Pathways and gene-pathway membership	298

^a^As source data elements do not correspond in a 1-to-1 manner with entities in the graph (eg, entities may be merged, filtered, or used as edges rather than nodes), actual counts for entities in AlzKB stratified by source are not available. The sizes are the best available estimates at the time of publication. Table 2 and Table S1 in Multimedia Appendix 1 [50-56] provide actual node and edge type counts in AlzKB.

^b^AOP-DB: Adverse Outcome Pathway Database.

^c^The derived data are structured in part using Hetionet.

^d^AD: Alzheimer disease.

^e^EPA: Environmental Protection Agency.

^f^DSSTox: Distributed Structure-Searchable Toxicity.

^g^ACToR: Aggregated Computational Toxicology Resource.

^h^GWAS: genome-wide association studies.

^i^LINCS: Library of Integrated Network-Based Cellular Signatures.

^j^NCBI: National Center for Biotechnology Information.

^k^MeSH: Medical Subject Headings.

^l^SIDER: Side Effect Resource.

^m^Counts not applicable (TISSUES associations map to edges rather than nodes in the graph).

**Table 2:** Node types and counts in the Alzheimer’s Knowledge Base listed in descending order by prevalence. Additional node types will be added over time, and counts will increase as new data sources are incorporated or existing sources are updated to newer versions.

Node label	Total nodes, N
Gene	62,407
Drug	35,063
BiologicalProcess	11,381
Pathway	4570
MolecularFunction	2884
CellularComponent	1391
Symptom	438
BodyPart	402
DrugClass	345
Disease	20

### Validating AlzKB Using Real-World Use Cases

After building AlzKB’s knowledge graph, we designed two ML-based use cases that resemble real-world tasks for which AlzKB was originally designed: (1) proposing genetic targets for new drugs based on disease similarity and topological graph features and (2) predicting new edges in the knowledge graph linking AD to repurposed drugs via a graph completion model. These 2 use cases are intended to assess the external validity of AlzKB—for the ML models to perform well on tasks defined using real-world evaluation end points (eg, effective drugs or etiologically important genes), the informative patterns and phenomena underlying those end points need to be adequately captured in the knowledge graph.

In the first use case (identifying genetic targets via graph topology measures), we trained a random forest (RF) classifier (implemented in the scikit-learn library [Python Software Foundation] for the Python programming language) using the following topological graph features, which are computed for every node pair in the graph (regardless of whether an edge does or does not exist between them): common neighbors, total neighbors, preferential attachment, Adamic-Adar, and resource allocation [57-60]. Each feature gives a different measure of network “relatedness” for a pair of nodes, which are then used as predictive features in the RF model. For a given node pair (*n*_1_, *n*_2_), these metrics are defined as follows:



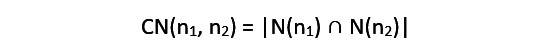


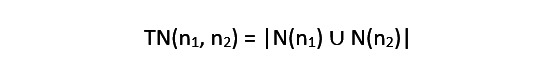


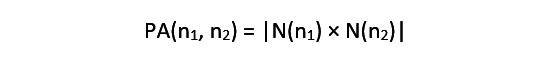


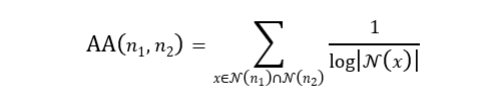


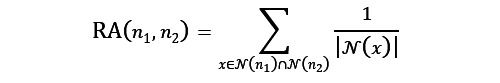



where N(n_1_) is the set of neighbor (adjacent) nodes of node *i*. Our training procedure for the RF model included 3-fold grid search cross-validation to optimize hyperparameters, an 80%/20% train/test split, and repeating the procedure 10 times with random sampling.

To accomplish the second use case (drug repurposing via graph completion models), we implemented and compared the performance of 5 graph completion algorithms applied to the entire AlzKB knowledge graph. These models learn low-dimensional representations of graph nodes as vector embeddings. The embeddings are then combined to propose all possible triples in the graph (source node, edge, and target node), and scores are generated to indicate the plausibility of the triple. The 5 models we evaluated are TransE, RotatE, DistMult, ComplEx, and ConvE [60].

We implemented the 5 models using PyKEEN—a Python library for knowledge graph embeddings [50]. We randomly split the data set of all triples into 80/10/10 training/validation/testing sets and used grid search to empirically set embedding dimensions to 256 and the number of epochs to 100 with early stopping allowed. All remaining hyperparameters were set to the PyKEEN defaults. We trained the models on Google Colab using a single Tesla T4 graphics processing unit and evaluated the results using the rank-based evaluation metrics hits@k (*k*=1, 3, and 10) and mean reciprocal rank (MRR) [61]. Ranking-based evaluation sorts the scores of triples in descending order and sets their rank as the index in the sorted list. In the case of multiple “true” triples having an equal score, we used the average of the most optimistic (best) and pessimistic (worst) ranks across the metrics. Briefly, hits@k is the ratio of true triples in the test set that have been ranked within the top *k* predictions of the model. Higher values indicate better performance. The MRR, also known as inverse harmonic mean rank, is the arithmetic mean of the inverse rank of the true triples. We performed evaluation on both left- and right-side predictions (ie, how well they can predict missing entities in partial triples without either the head [source] or tail [target] entities).

### Ethical Considerations

No human participants were involved in this research. All data used to build and evaluate AlzKB were derived from publicly available biomedical knowledge retrieved from open access databases. None of the data included were derived from individual human participants. Similarly, AlzKB is entirely open source and publicly available and complies with the licensing terms of all 22 source databases used to build the knowledge base.

## Results

### Knowledge Base Description

The first release of AlzKB (version 1.0) [26] contains 118,902 distinct nodes (representing biomedical entities) and 1,309,527 relationships linking those nodes. A full summary of node and relationship types with counts, respectively, is provided in Table 2 and Table S1 in Multimedia Appendix 1. Users can interact with AlzKB in their web browser using the built-in Neo4j interface or programmatically by connecting to the graph database over the internet. We also provide instructions for installing a local copy of the graph database as well as how to build the database from its original data sources.

### Proposing New Therapeutic Targets for AD

**Figure 2:**
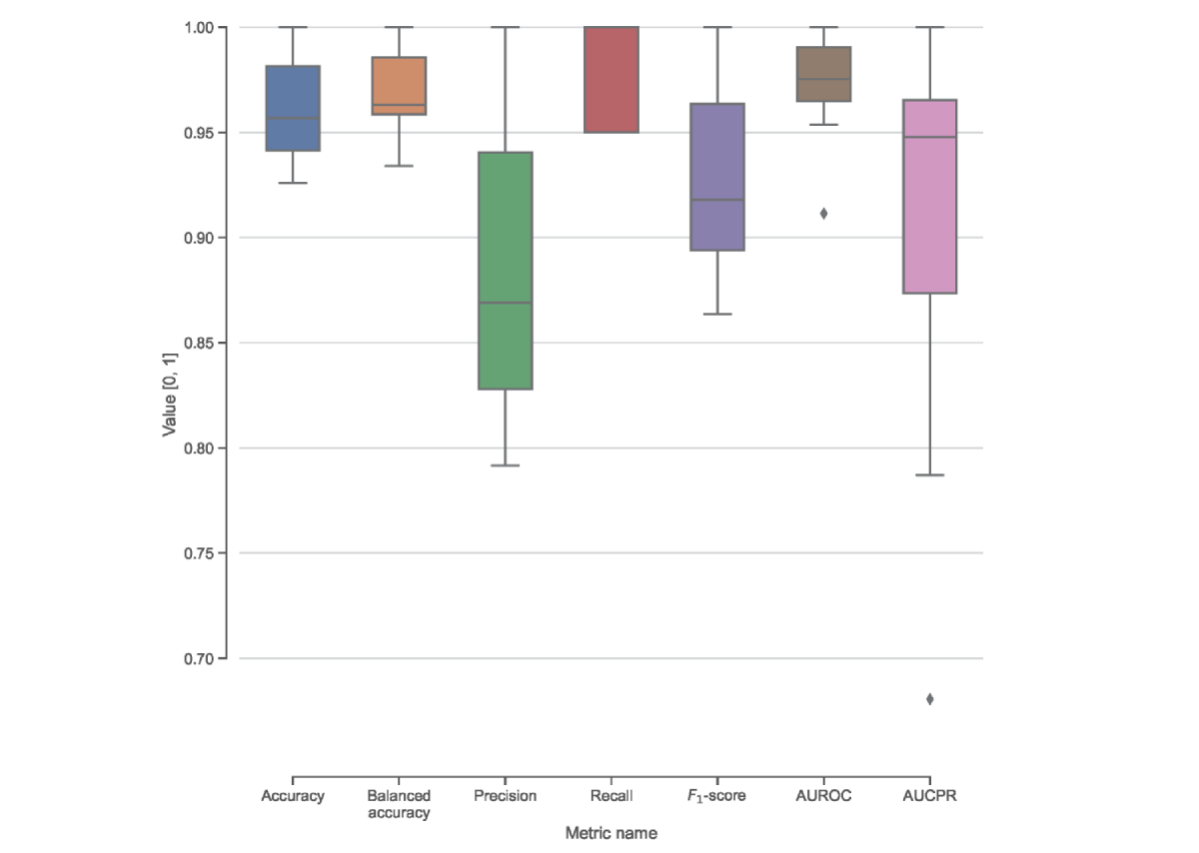
Random forest classifier performance (over 10 independent training runs) on the task of predicting whether Parkinson disease genes are also Alzheimer disease genes based on patterns of graph connectivity in the Alzheimer’s Knowledge Base’s heterogeneous knowledge graph. Across all metrics, a score of 1.00 represents the best possible performance. AUCPR: area under the precision-recall curve; AUROC: area under the receiver operating characteristic curve.

As a proof of concept, we performed an analysis to predict whether known PD genes are also linked to AD etiology. PD is a chronic, progressive neurological disorder characterized by uncontrollable movements and possible mental and behavioral changes. Similar to AD, the precise etiology of PD is not fully understood, but the disease is characterized by the death or dysfunction of basal ganglia neurons. A growing body of work has established physiological and genetic similarities between PD and AD [62], and it has been proposed that drugs targeting PD genes could potentially treat AD as well. To approach this hypothesis computationally, we defined a binary classification task to predict whether gene nodes in the AlzKB knowledge graph are or are not AD genes [63]. To assemble the data set, we considered all gene nodes adjacent to AD as positive (n=101) and all gene nodes not adjacent to AD as negative (n=62,306). The negative samples are assumed to contain a mixture of true negatives and false negatives; in link prediction tasks, the goal is to recover the false negatives. We further filtered the negative nodes to omit PD genes (n=73) and orphan gene nodes (n=43,032) and down sampled the remaining genes to 303 (ie, 3 times the number of positive samples). To evaluate the performance, we used accuracy, balanced accuracy, precision, recall, *F*_1_-score, area under the receiver operating characteristic curve, and area under the precision-recall curve, as shown in Figure 2.

The RF model predicted gene-disease relationships with an average balanced accuracy of 96.2% (precision=0.88; recall=0.98). We applied the trained models to predict PD genes that are likely to also be AD genes. Of the 73 PD genes in AlzKB, 8 (11%; *FYN*, *DCTN1*, *SNCA*, *SYNJ1*, *RSP12*, *ATXN2*, *KCNIP3*, and *CHRNB1*; described in Table 3) were predicted to be AD genes. A total of 10% (7/73) of the genes were predicted to be AD genes in all 10 models, whereas *CHRNB1* was predicted in 7 of the 10 models.

**Table 3:** Parkinson disease genes predicted by a graph-augmented random forest model to also be associated with Alzheimer disease.

Gene symbol	Gene name	Notes (from the Entrez Gene summary)
ATXN2	Ataxin-2	Modulates endocytosis, ribosomal translation, and mitochondrial function; aberrations are linked to diverse neurodegenerative diseases, diabetes, and obesity
CHRNB1	Cholinergic receptor nicotinic β1 subunit	Beta subunit of muscle acetylcholine receptor involved in transmitting signals at neuromuscular junction
DCTN1	Dynactin subunit 1	Dynactin is a macromolecular complex involved in many cellular functions, including the formation of neuronal pathways
FYN	FYN proto-oncogene, Src tyrosine kinase family	Membrane-associated tyrosine kinase involved in control of cell growth; highly expressed in brain tissue
KCNIP3	Potassium voltage-gated channel interacting protein 3	Voltage-gated potassium channel–interacting protein that is critical to neuronal excitability

### Drug Repurposing via Graph Data Science

As a second use case, we considered the task of repurposing existing drugs—currently used to treat other diseases—based on patterns in the knowledge graph that suggest that they may also treat AD. To do this, we trained 5 state-of-the-art knowledge graph completion methods (TransE, RotatE, DistMult, ComplEx, and ConvE) [51] on AlzKB and selected the highest-performing one to predict links between drugs and AD. Additional details about the differences between these methods are provided in Multimedia Appendix 1.

**Figure 3:**
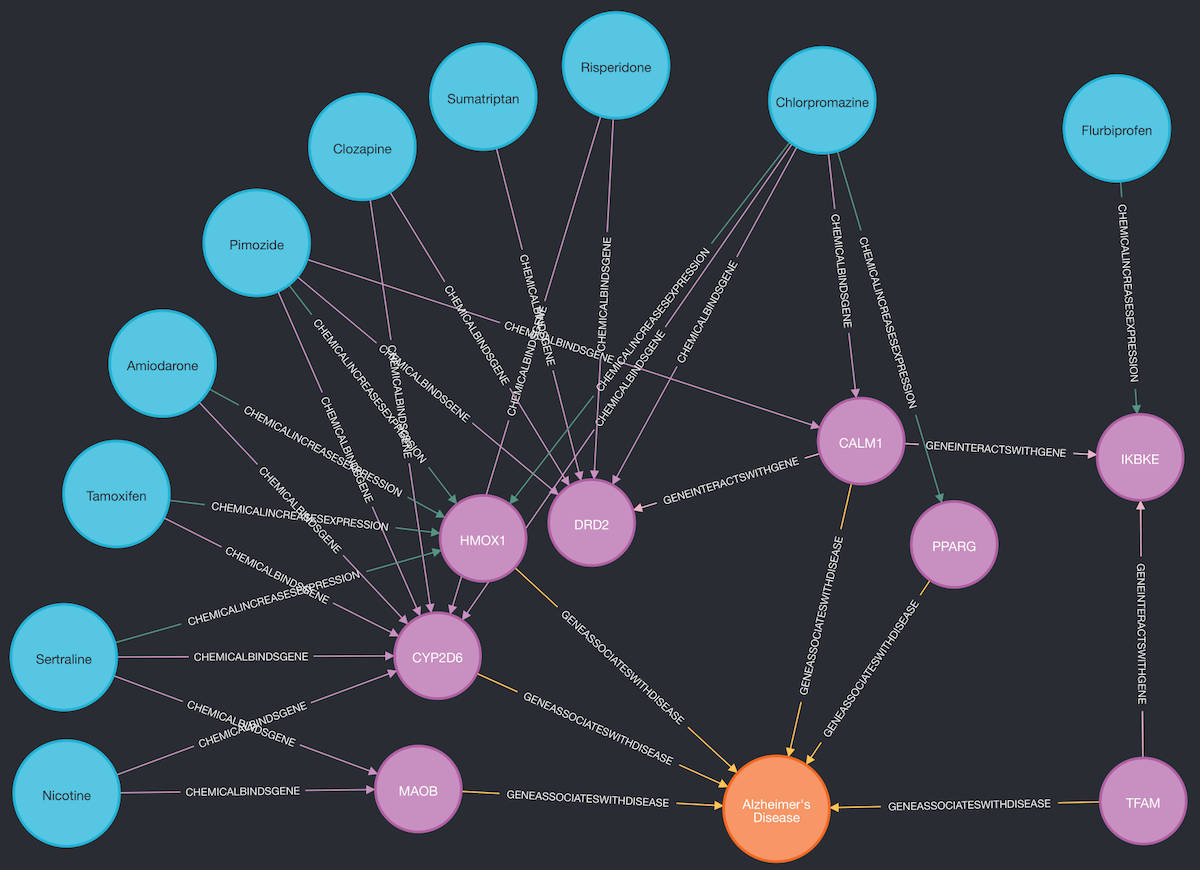
Spanning tree linking the 10 highest-scoring Alzheimer disease (AD) drug predictions (listed in Table 5) to AD. Blue nodes are drugs, pink nodes are genes, and the orange node is AD. Genes on the shortest path between a drug and AD can be considered putative mechanistic explanations for how the drug may act on AD etiology.

The performance of the 5 different knowledge graph completion models is shown in Table 4. Among them, RotatE performed best, with the highest MRR and hits@k values. Therefore, we used RotateE to make predictions on the test set to obtain missing head entities with the template ([*drug*], DRUG_TREATS_DISEASE, AD). The top 10 predicted drugs are listed in Table 5 along with their current approved use and relevant clinical trial status pertaining to AD efficacy. Of the top 10 predictions, 3 (30%) have been investigated in clinical trials to treat symptoms of AD. To further explore these predictions, we generated visualizations of a minimum spanning tree linking the 10 drugs to AD in AlzKB’s knowledge graph, as shown in Figure 3. The visualization shows that the shortest paths between the drugs and AD are mediated by a small set of AD-associated genes, each of which is associated with one or more of the proposed drugs. The visualization is suggestive of interpretable biological mechanisms through which the drugs could act on AD etiology and provides hypotheses to further explore their validity.

**Table 4:** Ranking-based evaluation metrics of 5 embedding-based link prediction models on the Alzheimer’s Knowledge Base knowledge graph. Metrics are derived from the likelihood of existing (known) links being predicted by the models. Higher scores indicate better performance.

Model name	Hits@1	Hits@3	Hits@10	MRR^a^
RotatE	*0.126* ^b^	*0.220*	*0.358*	*0.202*
TransE	0.046	0.097	0.198	0.097
DistMult	0.027	0.056	0.126	0.061
ComplEx	0.074	0.142	0.263	0.136
ConvE	0.002	0.005	0.013	0.006

^a^MRR: mean reciprocal rank.

^b^Italicized values indicate maximum scores within a given column.

**Table 5:** Drug repurposing predictions made by the best-performing topological link prediction model (RotatE). Also shown are current approved indications and (if available) clinical trials investigating the efficacy of the drug for treating Alzheimer disease (AD).

Drug name	Approved indications	AD-related clinical trials
Sumatriptan	Migraines and cluster headaches	—^a^
Nicotine	Nicotine withdrawal symptoms	NCT00018278 (completed)
Pimozide	Tourette disorder	—
Risperidone	Schizophrenia, bipolar mania, and psychosis	NCT00034762 (completed)
Flurbiprofen	Osteoarthritis and rheumatoid arthritis	—
Sertraline	Depressive disorder and social anxiety disorder	NCT00086138 (completed); NCT00009191 (completed)
Clozapine	Schizophrenia	—
Tamoxifen	ER+^b^ breast cancer	—
Amiodarone	Recurrent hemodynamically unstable ventricular tachycardia and recurrent ventricular fibrillation	—
Chlorpromazine	Nausea, vomiting, preoperative anxiety, schizophrenia, bipolar disorder, and severe behavioral problems in children	—

^a^No known AD-related clinical trials for the given drug.

^b^ER+: estrogen-receptor positive.

## Discussion

### Principal Findings

AlzKB is a freely available resource for the biomedical research community, with the primary goal of expanding the repertoire of therapies for AD via drug repurposing. In the previous sections, we described the current contents of AlzKB, the process of constructing it, and 2 specific data-driven use cases that illustrate how it can be applied to drug repurposing tasks. These use cases consisted of predicting the shared genetic architecture of AD and PD (potentially allowing for PD therapies to be repurposed for AD) and directly proposing drugs to repurpose for treating AD by predicting new links between drug and disease nodes in the knowledge graph. In both cases, the results are both biologically plausible and supported by quantitative metrics, yielding new hypotheses that merit experimental validation. AlzKB is a flexible resource that is not limited to these analyses, and we encourage other research teams to use it for different and complementary knowledge discovery tasks.

### The Role of AlzKB in Biomedical Knowledge Discovery

AD and other neurodegenerative diseases present one of the greatest challenges in modern biomedicine. AD is by and large a disease of old age, and as improvements to health care continue to increase the overall global life expectancy, we can expect the number of people with various forms of dementia to also increase. As the etiology and pathophysiology of AD are highly multifactorial, there is likely no single “cure” for the disease. Instead, researchers and public health officials have shifted much of their focus toward finding therapies that reduce risk, slow the progression of the disease, or reverse neuronal damage. In addition, as there are various subtypes of AD with underlying mechanisms, any therapy might be effective for only some patients with AD. Therefore, an essential step for reducing global disease burden is to propose many new therapeutic agents that target various aspects of AD pathology. This is precisely the motivating use case for AlzKB. As we have demonstrated, AlzKB provides a rich representation of existing knowledge about AD and the biological context in which it acts. The 2 ML-based use cases we presented previously use real-world end points to demonstrate that the knowledge captured in AlzKB is meaningful and representative of the biological processes underlying the disease. AlzKB stands to become a major resource in the AD research community, where pattern analysis and integration with observational data can be used to propose a diverse array of new therapeutic hypotheses along with interpretable mechanistic explanations of how those therapies may act in the human body.

Building the initial release of AlzKB was a highly interdisciplinary effort involving contributions from experts in translational bioinformatics, data science, and clinical informatics as well as medical scientists. Although each of these domains was essential in delivering a knowledge base that reflects important biomedical patterns describing AD etiology and treatment, a key need during the design and implementation phases was data literacy. To support future work in this and related areas, we encourage the inclusion of informatics and data analysis techniques in all types of biomedical curricula. Beyond AlzKB, our approach for building the knowledge graph is generalizable to practically any domain and depends on (1) defining an ontology using expert knowledge that formally describes the domain of interest and (2) identifying source databases that provide the entities and relationships described in the ontology. We are directly involved in the ongoing development of other knowledge bases using this same approach, including ComptoxAI—a knowledge base that supports AI research in toxicology [64]. As both knowledge bases share many of the same “core” entities (genes, diseases, pathways, and anatomical structures), the knowledge graphs are already semantically harmonized and ready for integration in larger, cross-disciplinary biomedical knowledge applications.

### Discovering Putative Therapies Through Graph Data Science

Of the PD genes predicted to also be AD genes (see the *Proposing New Therapeutic Targets for AD* section; Table 3), some are involved in neuronal signaling and structure, and some are known to be involved in a wide range of neurological disorders. *FYN* has seen recent attention and investigation into its possible link to AD due to its broad expression in brain tissue and known interactions with tau proteins [65,66]. Among the other identified genes, one (*CHRNB1*) is known to be involved in acetylcholine signaling [67,68], and another (*KCNIP3*) codes a protein that interacts with presenilin, and mutations in presenilin are causal for hereditary AD [69,70]. Some of these gene hits (*ATXN2* and *DCTN1*) have limited or no current research directly linking them to AD but are biologically plausible. As such, they may represent novel therapeutic targets or targets for further research and investigation [71]. For example, *DCTN1* encodes the dynactin-1 protein, and deficits in dynactin are connected to several neurodegenerative diseases; however, there is limited research linking this gene to AD [72,73].

Among the drug repurposing predictions (see the *Drug Repurposing via Graph Data Science* section; Table 5) are some agents that have previously been proposed for the treatment of AD (risperidone and sertraline) or for symptoms associated with AD (nicotine). Sumatriptan has been the subject of several studies focused on AD [74] and is connected to a strong comorbidity of migraine headaches and dementia in women [75]. Pimozide has been shown to reduce the aggregation of tau protein in mice [76] and is linked to AD in a number of unrelated *in silico* models [77]. The inclusion of nicotine is also noteworthy as it has seen recent interest among AD researchers and is the subject of an ongoing clinical trial to improve memory [78]. Other drugs listed in Table 5 have not yet been identified as AD treatments and represent novel repurposing candidates. Each can be considered a testable hypothesis meriting further investigation, giving credence to the increased detective power of AlzKB’s knowledge graph approach over existing AD data resources. It should be noted that this approach can only propose new indications for existing drugs and is based on existing knowledge and derived from known biological associations with those drugs. Other approaches (including emerging techniques in graph ML) could be used to propose entirely new drugs that could treat AD.

### Future Directions With AlzKB

AlzKB is a growing resource, and we have plans for adding new features and data types that are in various stages of implementation. As a central hypothesis of AD pathogenesis revolves around the atypical accumulation of proteins within and around brain cells, an important step will be to adequately distinguish and differentiate genes from the proteins that those genes code for. Existing data resources available for inclusion in AlzKB largely fail to make this distinction in a way that is accepted by the scientific community, so we are currently evaluating options to use either postprocessing of existing knowledge sources or synthesis of new knowledge to achieve a good representation of genes, proteins, and functional or structural variants that are key to understanding AD.

Current ML models often do not generalize well to heterogeneous graphs such as the one that constitutes AlzKB’s knowledge graph. This is largely because traditional models cannot use the network structure and heterogeneous nature of different entity types. Several promising algorithms can be used for prediction on heterogeneous graphs—including GraphSAGE [79] and metapath2vec [80]—but most fail to scale effectively when the number of node or edge types increases. As any effective therapy must be accompanied by a mechanistic understanding of how it functions, we also need to ensure that new heterogeneous graph ML models are *explainable*. With this in mind, we are using AlzKB as a motivating resource for designing new, cutting-edge algorithms that produce interpretable predictions from highly heterogeneous knowledge graphs. Furthermore, the increasing popularity of large language models (LLMs; such as GPT-4) presents a wealth of opportunities for incorporating knowledge graphs such as AlzKB into diverse AI applications [81]. One application we are considering is using AlzKB to provide LLMs with formalized knowledge about AD that allows them to more effectively produce informative outputs about AD etiology. Currently, LLMs can perform poorly on technically complex or poorly understood domains due to a scarcity of relevant content in their training corpora, and augmenting their performance using domain-specific knowledge graphs is an emerging strategy for fixing that issue. As we do so, these will be released alongside AlzKB with educational resources that facilitate ease of use and adoptability by various stakeholders.

Knowledge graphs—including AlzKB—come with several important limitations that will be crucial to address in coming years. One of these is the subjective nature of determining what does and does not constitute “knowledge,” implying broad acceptance by the scientific community (as opposed to “data,” which consist of individual observations). Currently, we use expert domain knowledge and careful screening of source databases to accomplish this, but with the advent of broadly accessible generative AI tools, there may be emerging strategies that minimize sources of human bias [82]. Furthermore, new predictions made using knowledge graphs still necessitate costly and time-consuming experimental or observational follow-up studies to validate those predictions. This is due in part to the absence of negative samples for training predictive models. While the presence of an edge between 2 nodes in a knowledge graph is interpreted as a “positive sample” for model training, the absence of an edge simply means that we do not know whether a relationship does or does not exist, and therefore, it may not in fact be a negative sample. New methods, including self-supervised contrastive learning, show promise in alleviating this issue [83], but further work is needed to determine whether these generalize well to AlzKB and similar highly heterogeneous biomedical knowledge graphs. Nonetheless, these are active areas of research in the AI, informatics, and computer science communities, and in spite of them, our results are still robust enough to provide compelling evidence demonstrating AlzKB’s scientific value.

Ultimately, we aim to provide AlzKB as a robust resource that helps unravel the etiology of AD. It is already a large, high-quality knowledge base from which graph-based AI or ML approaches can be developed for drug repurposing and drug discovery. As we and the rest of the biomedical research community make these discoveries in the coming years, they will be included and publicized on the AlzKB website as a public resource to drive innovation and scientific progress.

### Obtaining AlzKB for Local Use and Extending the Knowledge Graph

As it is a public and open-source resource for scientific discovery, we provide AlzKB through a variety of interfaces with distinct advantages for different use cases and user types. Casual users who wish to browse the knowledge base or perform simple analyses can do so directly through the Neo4j browser interface [24]. However, for more advanced use cases (or when computational needs exceed those available on the public version of the knowledge base), AlzKB can be either downloaded and populated locally into a Neo4j installation or built from the original source data files via the tools included on the AlzKB GitHub repository [25]. The latter of these options also allows users to extend the knowledge base to include additional data sources, entity types, or relationships beyond those provided in the official knowledge base distribution. We also encourage users who make modifications to the knowledge base to submit their changes for review to be included in the main code distribution. Instructions for how to contribute to AlzKB are also available on the GitHub repository.

As the data sources included in AlzKB are all, themselves, from open-source databases, we urge users to ensure that any new data sources they merge into AlzKB similarly comply with open-source standards. In brief, AlzKB can only be maintained under the most restrictive license terms of its included third-party sources, so restrictive license terms in a database being considered decrease that database’s suitability for inclusion. We hope for AlzKB to be recognized as a community effort for aggregating and democratizing the discovery of new AD therapeutics and, therefore, encourage public discussion of new methods and data sources to be included.

### Conclusions

In this work, we introduced AlzKB as a free, publicly available toolkit and data resource for novel discoveries in AD research, with a particular focus on therapeutic approaches to treating AD. AlzKB is both new and continually growing, and we aim to cultivate a community of researchers to collaboratively increase the impact, speed, and throughput of AD research, along with rapid dissemination to health care, academia, and the pharmaceutical industry. In the future, we will develop new AI and data science methods to continually extract knowledge from AlzKB, but in this study, we already demonstrate through graph data science that AlzKB can both replicate existing AD knowledge and generate entirely new, testable hypotheses to drive the future of drug repurposing and drug discovery.

## Appendix

Multimedia Appendix 1 Supplemental information providing expanded details on the knowledge graph completion methods used to validate Alzheimer’s Knowledge Base, as well as counts for relationship types in the knowledge graph.

## Abbreviations

AD: Alzheimer disease
AI: artificial intelligence
AlzKB: Alzheimer’s Knowledge Base
LLM: large language model
ML: machine learning
MRR: mean reciprocal rank
OWL: Web Ontology Language
PD: Parkinson disease
RDF: Resource Description Framework
RF: random forest

## Data Availability

The data sets generated during and analyzed during this study are available in the GitHub and Zenodo repositories [25,26].
