# Supplementary material for: The Alzheimer’s Knowledge Base: A Knowledge Graph for Alzheimer Disease Research
Source: J Med Internet Res. 2024 Apr 18;26:e46777. doi: 10.2196/46777 (PMC11066745; doi:10.2196/46777)
Supplement: Multimedia Appendix 1 [file jmir_v26i1e46777_app1.docx]

**Supplemental Information for “The Alzheimer’s Knowledge Base – A knowledge graph for therapeutic discovery in Alzheimer’s Disease research”**

**Authors**: Joseph D. Romano^1,2,3,*^, Van Truong^1,4,5^, Rachit Kumar^1,4,5,6^, Mythreye Venkatesan^7^, Britney E. Graham^7^, Yun Hao^1,4^, Nick Matsumoto^7^, Xi Li^7^, Zhiping Wang^7^, Marylyn Ritchie^1,3,5^, Li Shen^1,3^, Jason H. Moore^7,†^

**Author Affiliations**:
^1^ Institute for Biomedical Informatics, University of Pennsylvania, Philadelphia, PA
^2^ Center of Excellence in Environmental Toxicology, University of Pennsylvania, Philadelphia, PA
^3^ Department of Biostatistics, Epidemiology & Informatics, University of Pennsylvania, Philadelphia, PA
^4^ Graduate Group in Genomics and Computational Biology, University of Pennsylvania, Philadelphia, PA
^5^ Department of Genetics, University of Pennsylvania, Philadelphia, PA
^6^ Medical Scientist Training Program, University of Pennsylvania, Philadelphia, PA
^7^ Department of Computational Biomedicine, Cedars-Sinai Medical Center, Los Angeles, CA.

* Corresponding Author: Joseph.Romano@pennmedicine.upenn.edu
† Co-corresponding Author: Jason.Moore@csmc.edu

**Description of knowledge graph completion methods in §3.3**

In **§3.3** (**Drug repurposing via graph data science**), we trained 5 state-of-the-art knowledge graph completion methods for the task of repurposing existing drugs to treat AD based on graph topology. The techniques used by the different methods are diverse, but generally involve finding an embedded representation of the nodes and/or edges in the graph, and then use differences between the embeddings to infer whether an edge should exist between a given pair of nodes. Each of the 5 methods is implemented as part of the PyKEEN library,[1] as used in this study. Future work will involve the use of graph neural networks (GNNs, such as R-GCN, TransGCN, and others) which tend to better utilize complex graph structures due to their multi-layer belief propagation approach.

The following descriptions of the 5 completion methods we used provide a high-level overview of the approach, but for a more thorough comparison (including formal definitions) we refer the reader to Zamini *et al*.[2]

- **TransE**:[3] The most common translational distance model, where low-dimensional embeddings are computed and distance between those embedding vectors are used to calculate a score for possible links. TransE models both entities (nodes) and relations (edges) as vectors in the same embedding space, where the ‘head’ entity vector and the ‘tail’ entity vector are roughly different by the relation vector. Many modifications of TransE have emerged since its introduction.
- **RotatE**:[4] Another translational model, where relations are modeled as ‘rotations’ in a complex vector space (rather than translations, as in TransE). The rotation-based approach allows for easier detection of symmetric/antisymmetric, compositional, and inverse relationships.
- **DistMult**:[5] A tensor decomposition method, where relations are modeled as bilinear diagonal matrices rather than vectors (as in TransE and RotatE). A drawback of DistMult is that only symmetric relations can be inferred, due to its dependence on a diagonal matrix representation. Its major advantage is the low number of parameters learned per relation, resulting in a simpler (and possibly more interpretable) model.
- **ComplEx**:[6] Another tensor decomposition method that is a generalization of DistMult. ComplEx uses complex-valued embeddings, which allows for asymmetric relation modeling.
- **ConvE**:[7] A convolutional network-based approach that produces embeddings by feeding entities through a 2D convolutional layer, a projection layer that specifies the embedding dimension, and an inner product layer. This model is computationally and mathematically more simple than a true graph neural network, which means it is easier to train and implement, but attains lower performance due to its relatively shallow nature.

**Table S1 – Relationship types represented in AlzKB.**

| **Relationship Types** | **Total Edges** |
| --- | --- |
| GeneParticipatesInBiologicalProcess | 559,389 |
| GeneInPathway | 179,464 |
| GeneInteractsWithGene | 147,008 |
| BodyPartUnderexpressesGene | 102,194 |
| BodyPartOverexpressesGene | 97,782 |
| GeneHasMolecularFunction | 97,191 |
| GeneAssociatedWithCellularComponent | 73,553 |
| ChemicalDecreasesExpression | 21,051 |
| ChemicalIncreasesExpression | 18,713 |
| ChemicalBindsGene | 11,531 |
| DrugInClass | 1,029 |
| DrugTreatsDisease | 1,029 |
| SymptomManifestationOfDisease | 79 |
| DiseaseLocalizesToAnatomy | 29 |
| DrugCausesEffect | 2 |
| DiseaseAssociatesWithDisease | 1 |

**References**

1 Ali M, Berrendorf M, Hoyt CT, *et al.* PyKEEN 1.0: A Python Library for Training and Evaluating Knowledge Graph Embeddings. *Journal of Machine Learning Research* 2021;**22**:1–6.

2 Zamini M, Reza H, Rabiei M. A Review of Knowledge Graph Completion. *Information* 2022;**13**:396. doi:10.3390/info13080396

3 Bordes A, Usunier N, Garcia-Duran A, *et al.* Translating embeddings for modeling multi-relational data. *Advances in neural information processing systems* 2013;**26**.

4 Sun Z, Deng Z-H, Nie J-Y, *et al.* RotatE: Knowledge Graph Embedding by Relational Rotation in Complex Space. 2019.http://arxiv.org/abs/1902.10197 (accessed 2 Dec 2022).

5 Yang B, Yih W, He X, *et al.* Embedding Entities and Relations for Learning and Inference in Knowledge Bases. 2015.http://arxiv.org/abs/1412.6575 (accessed 2 Dec 2022).

6 Trouillon T, Welbl J, Riedel S, *et al.* Complex embeddings for simple link prediction. In: *International conference on machine learning*. PMLR 2016. 2071–80.

7 Dettmers T, Minervini P, Stenetorp P, *et al.* Convolutional 2D Knowledge Graph Embeddings. 2018.http://arxiv.org/abs/1707.01476 (accessed 2 Dec 2022).
